# Caffeoyl-Prolyl-Histidine Amide Inhibits Fyn and Alleviates Atopic Dermatitis-Like Phenotypes via Suppression of NF-κB Activation

**DOI:** 10.3390/ijms21197160

**Published:** 2020-09-28

**Authors:** Hayan Jeong, Jee Youn Shin, Kwanghyun Lee, Su-Jin Lee, Hyo-Jin Chong, Hyeri Jeong, Young-Eun Jeon, Dong-Sik Shin, Sunhyae Jang, Kyu Han Kim, Seok-In Kim, Yoon-Sik Lee, Bong-Gun Ju

**Affiliations:** 1 Department of Life Science, Sogang University, Seoul 04107, Korea; hayan90@sogang.ac.kr (H.J.); osiris98@sogang.ac.kr (J.Y.S.); khiston@sogang.ac.kr (K.L.); leesj@sogang.ac.kr (S.-J.L.); chd3695@sogang.ac.kr (H.-J.C.); 2 Department of Chemical and Biological Engineering, Sookmyung Women’s University, Seoul 04310, Korea; wpg@sookmyung.ac.kr (H.J.); jun24600@sookmyung.ac.kr (Y.-E.J.); dshin@sookmyung.ac.kr (D.-S.S.); 3 Laboratory of Cutaneous Aging and Hair Research, Clinical Research Institute, Seoul National University Hospital, Seoul 03080, Korea; jsh201@snu.ac.kr (S.J.); kyuhkim@snu.ac.kr (K.H.K.); 4 Institute of Human Environment Interface Biology, Seoul National University, Seoul 08826, Korea; 5 Department of Dermatology, College of Medicine, Seoul National University, Seoul 03080, Korea; 6 BeadTech Inc. 10-dong 4th, 49 Wonsi-ro, Danwon-gu, Ansan-si, Gyeonggi-do 15610, Korea; 1017sikim@beadtech.co.kr (S.-I.K.); yslee@beadtech.co.kr (Y.-S.L.)

**Keywords:** atopic dermatitis, CA-PH, Fyn, NF-κB, SYK, skin atrophy

## Abstract

Caffeic acid (CA) is produced from a variety of plants and has diverse biological functions, including anti-inflammation activity. It has been recently demonstrated that caffeoyl-prolyl-histidine amide (CA-PH), which is CA conjugated with proline-histidine dipeptide, relieves atopic dermatitis (AD)-like phenotypes in mouse. In this study, we investigated the molecular mechanism underlying CA-PH-mediated alleviation of AD-like phenotypes using cell line and AD mouse models. We confirmed that CA-PH suppresses AD-like phenotypes, such as increased epidermal thickening, infiltration of mast cells, and dysregulated gene expression of cytokines. CA-PH suppressed up-regulation of cytokine expression through inhibition of nuclear translocation of NF-κB. Using a CA-PH affinity pull-down assay, we found that CA-PH binds to Fyn. In silico molecular docking and enzyme kinetic studies revealed that CA-PH binds to the ATP binding site and inhibits Fyn competitively with ATP. CA-PH further suppressed spleen tyrosine kinase (SYK)/inhibitor of nuclear factor kappa B kinase (IKK)/inhibitor of nuclear factor kappa B (IκB) signaling, which is required for nuclear factor kappa-light-chain-enhancer of activated B cells (NF-κB) activation. In addition, chronic application of CA-PH, in contrast with that of glucocorticoids, did not induce up-regulation of regulated in development and DNA damage response 1 (REDD1), reduction of mammalian target of rapamycin (mTOR) signaling, or skin atrophy. Thus, our study suggests that CA-PH treatment may help to reduce skin inflammation via down-regulation of NF-κB activation, and Fyn may be a new therapeutic target of inflammatory skin diseases, such as AD.

## 1. Introduction

Caffeic acid (CA; 3,4-dihydroxycinnamic acid), which is a phenolic compound produced from a variety of plants, removes reactive oxygen species (ROS) by delocalization of the unpaired electron in the extended conjugated side chain [1,2]. The metal-chelating activity of CA also contributes to the antioxidant activity [3]. Previous studies indicated that CA has diverse biological effects on inflammation, carcinogenesis, and antibacterial activity [4,5,6]. In particular, CA, which is a major compound of water extract from *Ixeris dentata,* decreased expression of chemokines including IL-8 and TARC in keratinocytes and suppressed NF-κB activation through inhibition of p38 phosphorylation, resulting in reduction of the level of IL-8 and TNFα in mast cells [7].

**Figure 1:**
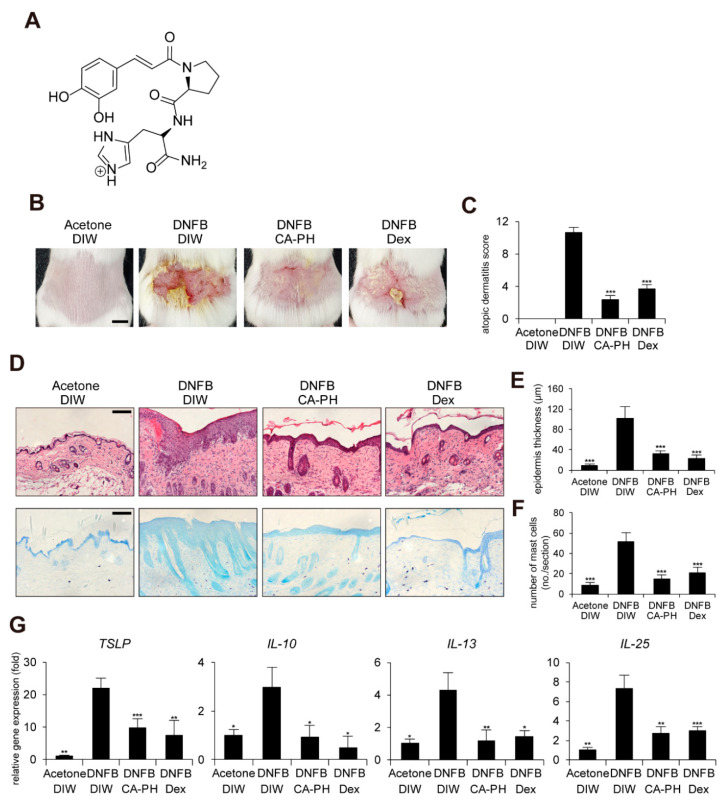
CA-PH alleviates AD-like phenotypes induced by DNFB treatment. Topical application of CA-PH alleviates AD-like phenotypes induced by DNFB treatment in mouse skin. Dex was used as the positive control. Acetone and DIW were used as solvents for DNFB and CA-PH, respectively. (**A**) Structure of CA-PH. (**B**) After mice were sensitized with DNFB for 7 days, DNFB was further topically applied to the shaved dorsal skin with or without CA-PH for 12 days (*n* = 6/group). Mice were photographed and skin tissues were harvested. Representative images are shown. Scale bar, 0.5 cm (**C**) CA-PH alleviates the severity of AD-like phenotypes induced by DNFB treatment in mouse skin. The severity of dermatitis was determined by a scoring index of AD (see Material and Methods). (**D**) Tissue sections from the back skin were stained with hematoxylin and eosin (H&E) or toluidine blue (TB). Representative images are shown. Scale bar, 50 μm. (**E**) CA-PH suppresses the increased epidermis thickness induced by DNFB treatment in mouse skin. Epidermis thicknesses were measured (*n* = 6/group). (**F**) CA-PH suppresses infiltration of mast cells induced by DNFB treatment in mouse skin. The number of mast cells was counted from five randomly selected low-power fields (*n* = 6/group). (**G**) CA-PH suppresses DNFB-induced up-regulation of cytokine expression in mouse skin. Transcripts of TSLP, IL-10, IL-13, IL-25, and RPLP0 from mouse skin were quantified using real-time PCR (*n* = 6/group). All data represent means ± S.E.M. Significance values were * *p* ≤ 0.05, ** *p* ≤ 0.01, and *** *p* ≤ 0.005. Dex, dexamethasone; DIW, deionized water.

In order to improve the chemical, physical, and biological properties of CA, caffeoyl-prolyl-histidine amide (CA-PH) was developed by conjugation of CA with proline-histidine dipeptide (Figure 1A). CA-PH has stronger antioxidant activity and enhanced stability [8,9]. CA-PH treatment efficiently reduced ROS generation and increased heme-oxygenase-1 expression in H_2_O_2_-treated vascular smooth muscle cells [9]. It has been recently reported that CA-PH relieves atopic dermatitis (AD)-like phenotypes in mouse models [10]. Jang et al. found that CA-PH reduces epidermal thickening, number of mast cells, and eosinophil infiltration, as well as recovery of dysregulation of genes related to the skin barrier and cytokines [10].

Fyn is a non-receptor tyrosine kinase belonging to the Src family kinases (SFKs), which include Blk, Brk, Fgr, Frk, Hck, Lck, Lyn, c-Src, Srm, and c-Yes in human [11,12]. Fyn transmits signals from diverse cell surface receptors to cytoplasmic signal transduction cascades [13]. It also plays important roles in the regulation of diverse biological functions, such as apoptosis, survival, adhesion, migration, neuronal transmission, and immunity [14,15,16,17]. Fyn contains an N-terminal region, two Src homology (SH) domains, a highly conserved catalytic domain including an adenosine triphosphate (ATP)-binding site, and a C-terminal tail, which negatively regulates tyrosine phosphorylation [18]. Autophosphorylation of the tyrosine 420 residue of Fyn is required for Fyn activation, which leads to tyrosine phosphorylation of a variety of substrates, including Tau, AMPK, and PIKE-A [17,19,20].

In skin, Fyn plays an important role in keratinocyte differentiation via regulation of cell–cell adhesion by tyrosine phosphorylation of β- and γ-catenin and p120^ctn^ [21,22,23,24]. Fyn has also been involved in keratinocyte transformation [25]. Expression of Fyn was up-regulated in cutaneous squamous cell carcinomas [26]. Overexpression of constitutively active Fyn (Y528F) in mouse epidermis induced keratotic tumors and actinic keratosis by down-regulation of the Notch1 and p53 and induction of the Ras/MAPK, PI3K/Akt, and STAT3 signaling pathways [27]. Fyn is involved in TNFα- or B[a]PDE (Benzo[a]pyrene-7,8-diol-9,10-epoxide)-induced up-regulation of COX-2, a key enzyme mediating inflammation, in JB6 P+ mouse epithelial cells [28,29]. In addition, the immunomodulatory function of Fyn has been reported in mast cells. For example, inhibition of Fyn suppresses IgE-mediated passive cutaneous anaphylaxis through inhibition of secretion of pro-inflammatory cytokines in mast cells [30]. Inhibition of Fyn also suppresses production and secretion of pro-inflammatory cytokines and IgE in 1-fluoro-2,4-dinitrobenzene (DNFB)-induced allergic dermatitis models, as well as inhibition of mast cell degranulation [31].

In this study, we investigated the molecular mechanism underlying CA-PH-mediated alleviation of AD-like phenotypes using cell line and AD mouse model. We found that CA-PH binds to and inhibits Fyn and suppresses NF-κB activation through inhibition of SYK/IKK/IκB signaling. 

## 2. Results

### 2.1. CA-PH Alleviates AD-Like Phenotypes Induced by DNFB Treatment

It has been recently shown that CA-PH treatment relieves 2,4-dinitrochlorobenzene (DNCB)-induced AD-like phenotypes in mice [10]. In this study, we further investigated the molecular mechanism of CA-PH-mediated alleviation of AD-like phenotypes using cell line and mouse models. We first examined the beneficial effect of CA-PH on mouse AD-like phenotypes induced by DNFB treatment. Dexamethasone was topically applied as a positive control. We measured the skin severity as a sum of 4 symptoms, namely hemorrhage, swelling, erosion, and dryness, using an AD scoring index (See Materials and Methods). CA-PH treatment alleviated DNFB-induced AD-like phenotypes (Figure 1B,C). Histological observations showed that CA-PH treatment suppresses DNFB-induced skin hyperplasia (Figure 1D,E). We also found decreased infiltration of mast cells by CA-PH treatment (Figure 1D,F). We further examined the expression of cytokines related to AD. CA-PH treatment suppressed gene expression of thymic stromal lymphopoietin (TSLP), interleukin-10 (IL-10), interleukin-13 (IL-13), and interleukin-25 (IL-25) in DNFB-treated mouse skin (Figure 1G).

### 2.2. CA-PH Suppresses Nuclear Translocation of NF-κB

**Figure 2:**
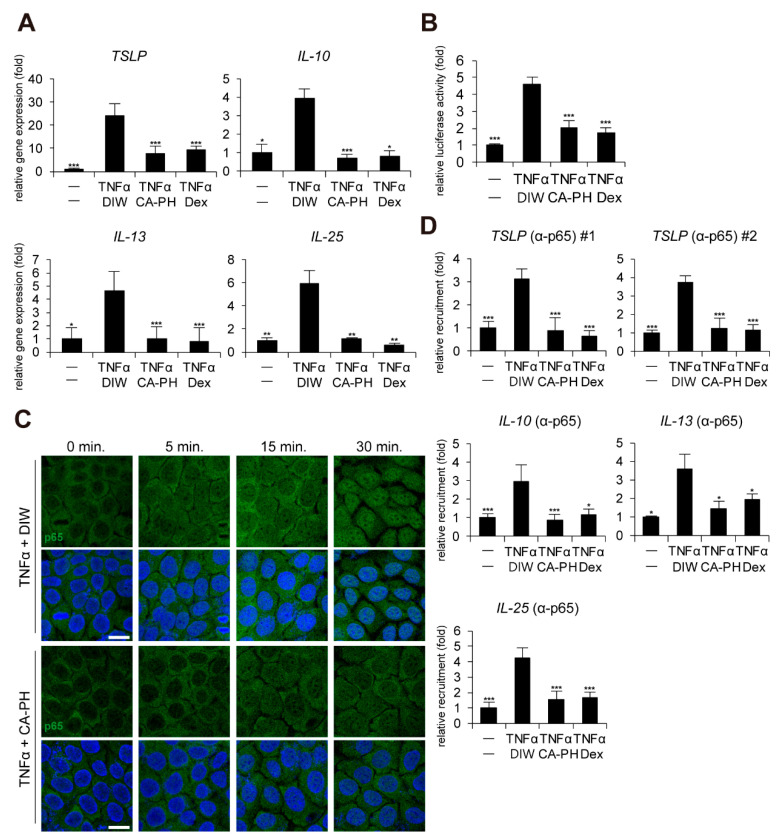
CA-PH inhibits NF-κB signaling in TNFα-treated keratinocytes. (**A**) CA-PH suppresses DNFB-induced up-regulation of cytokine expression in TNFα-treated HaCaT keratinocytes. Dex was used as the positive control. DIW was used as a solvent for TNFα and CA-PH. Transcripts of TSLP, IL-10, IL-13, IL-25, and RPLP0 were quantified using real-time PCR (*n* = 5). (**B**) CA-PH suppresses the activity of the luciferase reporter containing NF-κB binding sites in TNFα-treated HaCaT keratinocytes. Dex was used as the positive control. DIW was used as the solvent for TNFα and CA-PH. Following transfection of the luciferase reporter vector containing NF-κB binding sites and the control *Renilla* luciferase expression vector into HaCaT keratinocytes, luciferase activity was measured in cell extracts (*n* = 5). Reporter activity is represented as the fold activation relative to *Renilla* luciferase activity. (**C**) CA-PH inhibits nuclear translocation of NF-κB p65 in TNFα-treated HaCaT keratinocytes. Dex was used as the positive control. DIW was used as the solvent for TNFα and CA-PH. Cells were immunostained with anti-NF-κB p65 antibody (*n* = 5). Nuclei were identified using DAPI staining. Representative images are shown. Scale bar, 20 μm. (**D**) TNFα induces the occupancy of NF-κB p65 at the NF-κB binding sites of TSLP, IL-10, IL-13, and IL-25 gene promoters in HaCaT keratinocytes. However, CA-PH decreases the occupancy of NF-κB p65 in the gene promoters in HaCaT keratinocytes treated with TNFα. Dex was used as positive control. DIW was used as solvent for TNFα and CA-PH. ChIP assay was performed using anti-NF-κB p65 antibody (*n* = 5). The occupancy of each protein was quantified using real-time PCR in the gene promoters encompassing the NF-κB binding sites. All data represent means ± S.E.M. Significance values were * *p* ≤ 0.05, ** *p* ≤ 0.01, and *** *p* ≤ 0.005. Dex, dexamethasone; DIW, deionized water; TNFα, tumor necrosis factor alpha.

Given that keratinocytes, a major cell type in epidermis, contribute to AD pathogenesis and that up-regulation of cytokines including TSLP, IL-10, IL-13, and IL-25 is dependent on NF-κB activation in keratinocytes [32,33,34,35], we next investigated whether CA-PH modulates the NF-κB signaling pathway in HaCaT keratinocytes. CA-PH significantly suppressed up-regulation of TSLP, IL-10, IL-13, and IL-25 expression in TNFα-treated HaCaT keratinocytes (Figure 2A). In addition, CA-PH suppressed the activity of the promoter reporter containing NF-κB binding sites (Figure 2B).

Immunocytochemical examination indicated that CA-PH inhibits TNFα-induced nuclear translocalization of NF-κB p65 (Figure 2C). To confirm the inhibitory effect of CA-PH on NF-κB nuclear localization by TNFα treatment, ChIP was performed. As expected, NF-κB p65 occupied the NF-κB binding sites of the gene promoters of TSLP, IL-10, IL-13, and IL-25 in TNFα-treated keratinocytes. However, CA-PH suppressed the TNFα-induced occupancy of NF-κB p65 in the gene promoters (Figure 2D). 

### 2.3. CA-PH Binds to and Inhibits Fyn

**Figure 3:**
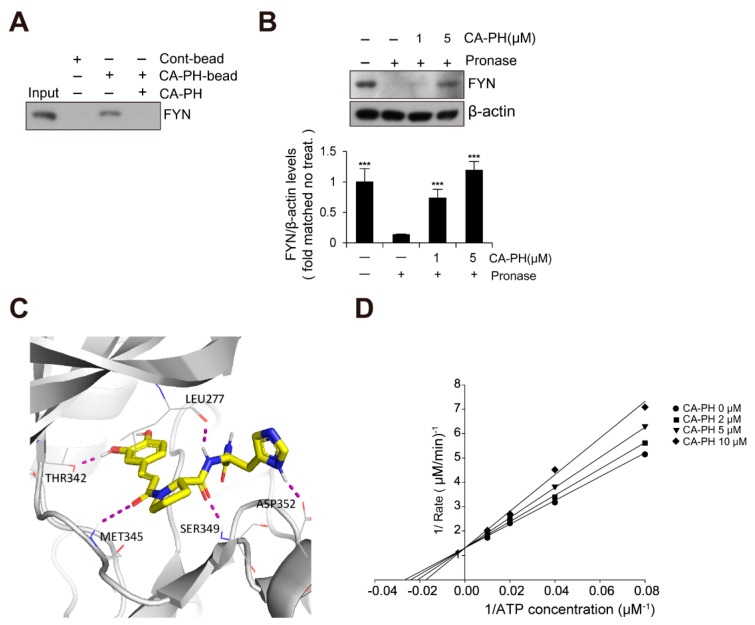
CA-PH binds to and inhibits Fyn. (**A**) CA-PH interacts with Fyn. CA-PH beads were prepared by binding of CA-PH to polystyrene beads (See Materials and Methods). CA-PH-bound beads were mixed with protein extract from HaCaT cells. After extensive washing, Western blotting was performed using anti-Fyn antibody. Free excess CA-PH was added as a competitor. (**B**) The drug affinity responsive target stability (DARTS) assay reveals that CA-PH-bound Fyn is resistant to protease digestion. Protein extract from HaCaT cells was mixed with CA-PH and treated with Pronase at room temperature. Western blot analysis was performed using anti-Fyn antibody. As a loading control, the reaction mixture without incubation was immunoblotted with anti-β actin antibody. Representative images are shown. Data were quantitatively assessed and are additionally depicted in graphs (*n* = 3). (**C**) Molecular docking shows that CA-PH binds to the ATP binding site of Fyn (∆*G* = −8.74 kcal/mol). CA-PH can form 5 hydrogen bonds with Leu277, Thr342, Met345, Ser349, and Asp352 residues within the ATP binding site of the catalytic domain. (**D**) CA-PH inhibits Fyn competitively with ATP. Double-reciprocal plots of the inhibitory activity of CA-PH. Fyn kinase activity was measured at the indicated concentrations of CA-PH and ATP (*n* = 3). The reciprocal velocity was plotted versus 1/(ATP). Km = 40.4 μM, Ki = 16 μM. Fyn kinase assay was performed using a Kinase Enzyme System. For enzyme kinetics, the Lineweaver–Burk method was applied. All data represent means ± S.E.M. Significance value was *** *p* ≤ 0.005.

Because Fyn has been previously identified as a binding molecule of CA [36], we tested whether CA-PH interacts with Fyn. We prepared CA-PH-bound beads by coupling CA-PH to polystyrene beads (see Materials and Methods). CA-PH-bound beads were mixed with protein extract from HaCaT keratinocytes. We found that Fyn interacts with CA-PH (Figure 3A). To further confirm CA-PH binding to Fyn, a DARTS assay was performed. Protein extract was mixed with CA-PH and incubated in the presence of Pronase, a mixture of nonspecific endo- and exo-proteases. CA-PH-bound Fyn was resistant to Pronase digestion, indicating that CA-PH binds to Fyn (Figure 3B). In silico molecular docking analysis (see Materials and Method) revealed that CA-PH may bind to the ATP binding site of Fyn with relatively high affinity (∆*G* = −8.74 kcal/mol) (Figure 3C). CA-PH can form 5 hydrogen bonds with Leu277, Thr342, Met345, Ser349, and Asp352 residues within the ATP binding site (Figure 3C). In particular, the CA moiety can form hydrogen bonds with Thr342 and Met345 residues in the hinge region [37,38]. Next, we tested whether CA-PH inhibits Fyn competitively or non-competitively with ATP. An enzyme assay was performed to determine kinetics using the Lineweaver–Burk plot method. Lineweaver–Burk plots for inhibition of Fyn by CA-PH with respect to ATP concentration showed all curves intersecting the y-intercept at zero, which is indicative of a competitive inhibition mechanism (Figure 3D). The value of Km (the Michaelis constant) is 40.4 µM and that of Ki (inhibition constant) is 16 μM in this reaction. Thus, the IC_50_ value was 43 µM.

Because c-Jun N-terminal kinase (JNK) and spleen tyrosine kinase (SYK) play important roles in AD pathogenesis [39,40,41,42], we further tested whether CA-PH also inhibits their activity. CA-PH did not inhibit the activity of JNK and minimally inhibited the activity of SYK (Supplemental Figure S1). In addition, PP2, a broad inhibitor for Src family kinases, including Fyn, suppressed the activity of the promoter reporter containing NF-κB binding sites (Supplemental Figure S2).

### 2.4. CA-PH Suppresses NF-κB Signaling Pathway by Inhibition of Fyn Activation

**Figure 4:**
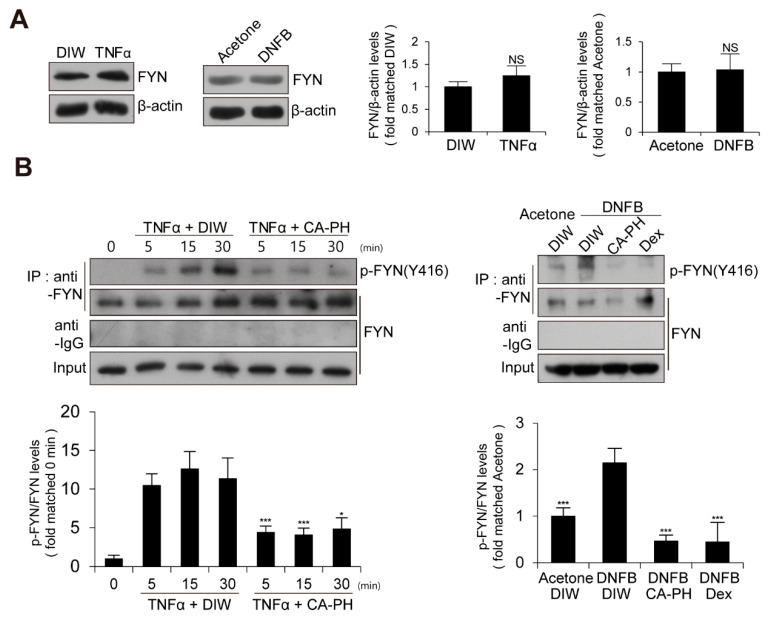
Activation of Fyn in TNFα-treated keratinocytes and DNFB-induced AD-like mouse skin. (**A**) Expression levels of Fyn are not changed in TNFα-treated HaCaT keratinocytes or DNFB-treated mouse skin. DIW and acetone were used as solvents for TNFα and DNFB, respectively. Western blot analysis was performed using anti-Fyn antibody. As a loading control, anti-β actin antibody was used. Representative images are shown. Data were quantitatively assessed and additionally depicted in graphs (*n* = 3). (**B**) Increased kinase activity of Fyn in TNFα-treated HaCaT keratinocytes or DNFB-treated mouse skin. Fyn was immunoprecipitated with anti-Fyn antibody and immunoblotted with anti-phospho SRC (Y416) antibody. Data were quantitatively assessed and are additionally depicted in graphs (*n* = 3). All data represent mean ± S.E.M. Significance values were * *p* ≤ 0.05, and *** *p* ≤ 0.005. Dex, dexamethasone; DIW, deionized water; TNFα, tumor necrosis factor alpha; NS, no significance.

We next examined the expression level and enzyme activity of Fyn in keratinocytes treated with TNFα or AD-like mouse skin induced by DNFB treatment. The expression level of Fyn was not changed (Figure 4A). To measure Fyn activity, we examined the phosphorylation level of Fyn at tyrosine residue 420 [12,43,44,45]. Fyn was immune-precipitated with anti-Fyn antibody and immunoblotted with anti-phospho SRC (Y416) antibody, which recognizes the phosphorylation of Fyn at tyrosine residue 420 [12,43,44,45]. Western blot analysis demonstrated that the activity of Fyn is increased in keratinocytes treated with TNFα or AD-like mouse skin induced by DNFB treatment (Figure 4A,B).

**Figure 5:**
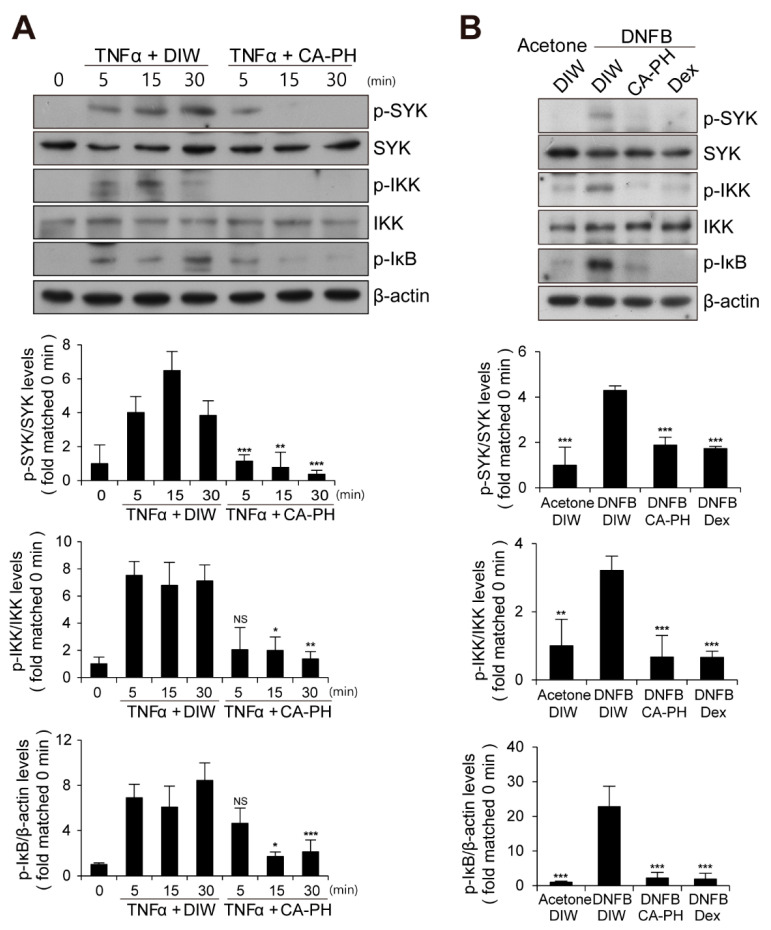
CA-PH suppresses NF-κB signaling in TNFα-treated keratinocytes and DNFB-induced AD-like mouse skin. (**A,B**) CA-PH inhibits phosphorylation of SYK, IKK, and IκB in TNFα-treated HaCaT keratinocytes or DNFB-treated mouse skin. Western blot analysis was performed using anti-SYK, anti-phospho SYK (Y525/526), anti-IKK, anti-phospho IKK (S176/177), and anti-phospho IκBα (S32) antibodies. As a loading control, Western blot analysis was performed using anti-β actin antibody. DIW was used as the solvent for TNFα and CA-PH. Acetone was used as the solvent for DNFB. Data were quantitatively assessed and are additionally depicted in graphs (*n* = 5). Representative images are shown. All data represent mean ± S.E.M. Significance values were * *p* ≤ 0.05, ** *p* ≤ 0.01, and *** *p* ≤ 0.005. Dex, dexamethasone; DIW, deionized water; TNFα, tumor necrosis factor alpha; NS, no significance.

Given that CA-PH inhibits nuclear translocation of NF-κB p65 in keratinocytes treated with TNFα (Figure 2C), we next examined the phosphorylation level of IκB, which inhibits nuclear translocation of NF-κB p65 when it is unphosphorylated. We found that CA-PH inhibits TNFα- or DNFB-induced phosphorylation of IκB in HaCaT keratinocytes or mouse skin (Figure 5A,B). We consistently found decreased phosphorylation of IKKα, an IκB kinase, by CA-PH treatment (Figure 5A,B). Because SYK, which has been reported as an AD target, acts as a downstream kinase of Fyn signaling and as an upstream kinase of IKK in several cell types [46,47], we further tested whether CA-PH inhibits SYK activity by examination of the phosphorylation level of SYK at tyrosine residues 525 and 526 [48,49,50]. TNFα or DNFB treatment induced phosphorylation of SYK in HaCaT keratinocytes or in mouse skin, respectively. However, CA-PH suppressed TNFα- or DNFB-induced phosphorylation of SYK (Figure 5A,B).

### 2.5. CA-PH Does not Induce Skin Atrophy

**Figure 6:**
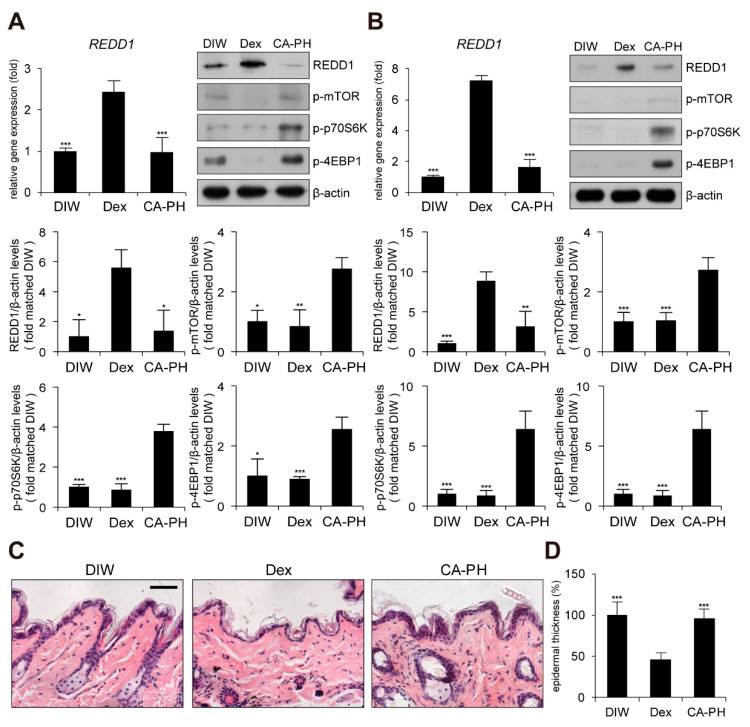
Chronic treatment of CA-PH does not induce skin atrophy. (**A**,**B**) Dex up-regulates regulated in development and DNA damage response 1 (REDD1) gene expression and inhibits mTOR signaling. However, CA-PH does not induce increased expression of REDD1 and inhibit mTOR signaling in HaCaT keratinocytes or mouse skin. Transcripts of REDD1 and RPLP0 were quantified using real-time PCR (*n* = 5). Western blot analysis was performed using anti-REDD1, anti-phospho mTOR (S2448), anti-phospho p70S6K (T389), anti-phospho 4EBP1 (S65), and anti-β actin antibodies. DIW was used as the solvent for CA-PH. Data were quantitatively assessed and are additionally depicted in graphs (*n* = 5). Representative images are shown. (**C**,**D**) Chronic application of dexamethasone (Dex) induces skin atrophy. However, CA-PH does not induce skin atrophy. Dexamethasone or CA-PH was treated for 1 week in mouse skin. DIW was used as the solvent for CA-PH. Tissue sections from the skin were stained with hematoxylin and eosin (H&E). Representative images are shown. Scale bar, 100 μm. Epidermis thicknesses were measured (*n* = 6/group). All data represent means ± S.E.M. Significance values were * *p* ≤ 0.05, ** *p* ≤ 0.01, and *** *p* ≤ 0.005. Dex, dexamethasone; DIW, deionized water.

Although glucocorticoids are effective for AD treatment by suppressing inflammation, chronic treatment with glucocorticoids induces adverse effects, such as skin atrophy, which is characterized by decreased skin thickness and elasticity, combined with a decreased barrier function [51,52,53,54]. Recent studies revealed that chronic glucocorticoid treatment induces skin atrophy through up-regulation of regulated in development and DNA damage response 1 (REDD1), an mTOR inhibitor [55]. Thus, we investigated whether chronic treatment with CA-PH induces skin atrophy by examining REDD1 expression and mTOR signaling. Dexamethasone elevated REDD1 gene expression and inhibited mTOR signaling by suppressing phosphorylation of mTOR, p70S6K, and 4EBP1 in HaCaT keratinocytes and mouse skin (Figure 6A,B). However, CA-PH did not activate REDD1 gene expression or inhibit mTOR signaling (Figure 6A,B). Histological examination further showed that chronic treatment of CA-PH did not reduce epidermis thickness compared with that of dexamethasone in mouse skin (Figure 6C,D).

## 3. Discussion

CA is found naturally in plants and exhibits various biological activities, including antioxidant and anti-inflammation activities [6,56,57]. To improve the chemical, physical, and biological properties of CA, CA-PH was developed by conjugation of CA with proline-histidine dipeptide [8,9]. CA-PH showed strong antioxidant activity and enhanced stability compared with CA [8,9]. Recently, Jang et al. reported that topical application of CA-PH relieves DNCB-induced AD-like phenotypes in mice [10]. They found that CA-PH reduced epidermal thickening, the number of mast cells, and eosinophil infiltration, while it recovered expression of skin-barrier-related proteins such as filaggrin, involucrin, and loricrin. It also decreased gene expression of IL-4, IL-6, IL-17a, IL-1b, IL-31, IL-33, and TSLP. In this study, we further confirmed a therapeutic effect of CA-PH in DNFB-induced AD-like phenotypes in mice. We also found that CA-PH suppresses up-regulation of cytokine gene expression through inhibition of nuclear translocation of NF-κB p65, resulting in decreasing recruitment of NF-κB p65 to its binding sites of TSLP, IL-10, IL-13, and IL-25 gene promoters.

It has been previously reported that CA binds to the ATP binding site and putative allosteric site between the SH2 domain and the kinase domain [36]. In the same study, molecular docking and biochemical analysis indicated that CA inhibits kinase activity non-competitively with ATP [36]. Using CA-PH affinity pull-down and a DARTS assay, we found that CA-PH binds to Fyn. The molecular docking predicted that CA-PH potentially binds to the ATP binding site in the catalytic domain with relatively high affinity (∆G= −8.74 kcal/mol). CA-PH could form 5 hydrogen bonds with Leu277, Thr342, Met345, Ser349, and Asp352 residues within the ATP binding site, possibly contributing to the high estimated binding energy. In particular, the CA moiety of CA-PH could form hydrogen bonds with Thr342 and Met345 residues in the hinge region. Because Met345 forms a hydrogen bond with Gly348 for hinge closing in the active state of Fyn, disruption of this bond results in hinge opening and the inactive state of Fyn [38]. Thr342 is a gatekeeper amino acid, which controls selectivity for small molecule inhibitors and helps anchor inhibitors to the active site [58,59]. Consistently, it has been reported that most Fyn inhibitors form hydrogen bonds with amino acid residues in the hinge domain, including Thr342 and Met345 [60]. Furthermore, the molecular docking predicted that CA-PH interacts with Fyn through Ser349 and Asp352 residues, which serve as anchor points in the ATP binding pocket [61]. Thus, CA-PH interaction with the ATP binding site in the catalytic domain may inhibit ATP-dependent kinase activity of Fyn [37,61]. In contrast to CA [36], an enzyme kinetics study demonstrated that CA-PH binds to the ATP binding site competitively with ATP. However, CA-PH did not inhibit JNK and minimally inhibited SYK, both of which are closely related to AD pathogenesis [62,63,64].

We found that Fyn activity, but not expression, is increased in TNFα-treated keratinocytes or AD-like mouse skin. Although it is not well known whether Fyn plays an important role in AD, a few reports suggest the protective role of Fyn in skin inflammation. For instance, dasatinib, an anti-cancer agent, suppressed the secretion of inflammatory cytokines by direct inhibition of Fyn in mast cells, which play an important role in type I hypersensitivity immune responses, including AD [65]. Similarly, *Rhamnus davurica* extract inhibited the activation of Fyn in antigen-stimulated mast cells [66]. Our results further indicate that Fyn activates NF-κB signaling in TNFα-treated keratinocytes or AD-like mouse skin. Similarly, lipopolysaccharide (LPS) induced an interaction between toll-like receptor 4 (TLR4) and Fyn resulted in activation of the PI3k/Akt/NF-κB pathway in primary astrocytes [67]. Constitutively active FynT (Y528F), an alternative splicing form in hematopoietic cells, induced highly increased pro-inflammatory cytokines and chemokines after prolonged TNFα treatment. However, these effects were abolished by broad Src-family kinase inhibitor PP2 and siRNA against FynT [68]. In addition, Fyn activity was critical for the initiation and progression of inflammatory signaling in adipose tissue [69]. Numerous Fyn inhibitors from plant extracts inhibited NF-κB activation. For example, saucerneol F and manassantin B from roots of *Saururus chinensis* inhibited the phosphorylation of Fyn and multiple downstream signaling processes, including the SYK/IKK/IκB pathway, resulting in inhibition of nuclear translocation of NF-κB p65 and cytokine expression in mast cells [46,47]. Similarly, cyanidin-3-glucoside directly bound to Fyn non-competitively with ATP and inhibited Fyn activity, which led to suppression of NF-κB activation in mouse skin epidermal JB6 P+ cells treated with benzo[a]pyrene-7,8-diol-9,10-epoxide (B[a]PDE) [29,70]. Myricetin, a flavonoid, acted as an ATP-competitive inhibitor to suppress Fyn activity and inhibited UVB-induced NF-κB activation in JB6 P+ cells and in mouse skin [70].

It is not clear currently how inhibition of Fyn activity by CA-PH contributes to inhibition of NF-κB activation. Our results demonstrate that Fyn may act as an upstream regulator of SYK/IKK/IκB signaling [46,47]. In particular, SYK has been investigated as a therapeutic target of skin inflammatory diseases such as AD [71,72,73,74]. ASN002, an oral dual JAK/SYK inhibitor, suppressed moderate to severe AD phenotypes in early clinical trials [41,42]. ASN002 reversed a lesional skin transcriptome toward a non-lesional phenotype [48]. It also suppressed key inflammatory pathways related to AD pathogenesis, such as T_H_2, T_H_17/T_H_22, and T_H_1 axis [41]. In addition, polydatin (3, 4′, 5-trihydroxystilbene-3-β-mono-D-glucoside), a natural component extracted from *Polygonum cuspidatum*, directly inhibited activity of SYK and suppressed pro-inflammatory cytokine expression in mast cells [75]. Extracts of *Cymbidium* *eburneo-kanran* and *Artemisia argyi* down-regulated the expression of pro-inflammatory cytokines through suppression of the signaling pathway, including phosphorylation of SYK [50,76,77]. The extracts further alleviated DNCB-induced AD-like phenotypes, including skin lesion severity, scratching behavior, and IgE levels [50,76,77].

We also found that chronic application of CA-PH, in contrast with that of glucocorticoids, does not induce skin atrophy. Although glucocorticoids efficiently suppress inflammation and are widely used to treat inflammatory skin diseases such as AD, chronic treatment often results in numerous adverse effects, including skin atrophy, causing profound losses of skin thickness and elasticity, combined with decreased barrier function [51,52,53,54]. In addition, REDD1, which is a stress-inducible mTOR inhibitor, has been identified as a major molecular target of glucocorticoids in skin atrophy [55,78,79]. Our results demonstrated that CA-PH does not induce up-regulation of REDD1 or inhibit mTOR signaling, resulting in no skin atrophy.

In conclusion, we identified the molecular mechanism underlying CA-PH-mediated alleviation of AD-like phenotypes. Specifically, we found that CA-PH binds to and inhibits Fyn, leading to suppression of NF-κB activation through reduction of SYK/IKK/IκB signaling. In addition, chronic application of CA-PH did not induce skin atrophy. Our study suggests that Fyn may be a new therapeutic target, and its inhibitors, including CA-PH, can be used as candidates for AD treatment. However, CA-PH is a moderate inhibitor of Fyn with a micromolar IC_50_ value compared with known active Fyn inhibitors with nanomolar IC_50_ values. Thus, CA-PH could be used as a lead compound, with modification required for development of an active Fyn inhibitor.

## 4. Materials and Methods 

### 4.1. Cell Culture

HaCaT keratinocytes were maintained in DMEM supplemented with 10% fetal bovine serum and 1% penicillin-streptomycin at 37 °C in a humidified atmosphere of 5% CO_2_. After cells were treated with 50 ng/mL TNFα (Sigma-Aldrich, St. Louis, MO, USA) for 1 h, cells were incubated with DIW, 1 µM CA-PH, 1 µM PP2 (Sigma-Aldrich, St. Louis, MO, USA), or 200 nM dexamethasone (Sigma-Aldrich, St. Louis, MO, USA) for 1 or 23 hr.

### 4.2. Mouse AD Model

Adult female BALB/c mice (6 weeks old, DBL, Korea) were maintained in a temperature-controlled room (22 °C) at 55% humidity, with a 12 h light–dark cycle. The Committee for Experimental Animal Research at Sogang University approved the animal experiments (code: IACUCSGU2017-2, date: 01 June 2017). Dermatitis was induced by DNFB (Sigma-Aldrich, St. Louis, MO, USA) in mice as described previously [80]. Briefly, the sensitization was performed once by topical application of 100 µL of 0.15% DNFB dissolved in acetone to the shaved abdominal skin of mice. A week later, the shaved dorsal skin areas of mice were topically treated with 100 µL of 0.15% DNFB every 3 days for 12 days. The same mice were also topically applied with 100 µL of DIW or 100 µL of 100 µM CA-PH daily for 12 days. In the negative control group, 100 µL of acetone or DMSO was topically applied to the shaved dorsal skin areas. As a positive control, DNFB-treated mice were topically treated with 100 µL of 200 µM dexamethasone.

### 4.3. Mouse Skin Atrophy Model

Skin atrophy was induced as previously described [81]. Briefly, 200 µL of DIW or 200 µL of 100 µM CA-PH was topically applied daily for 8 days to the shaved dorsal skin of adult female BALB/c mice (6 weeks old, DBL, Korea). As a positive control, 100 µL of 200 µM dexamethasone was applied for 8 days. Dorsal skin samples were harvested for further analysis.

### 4.4. Skin Lesion and AD-Like Phenotype Score

After mice were anesthetized with 2% isoflurane, skin lesions were photographed. The scores for the AD-like phenotypes were measured according to the criteria described previously, with slight modifications [82]. Total skin severity scores (maximum score: 12) were defined as 0 (none), 1 (mild), 2 (moderate), and 3 (severe) for each of the four symptoms: (i) erythema and hemorrhage, (ii) edema, (iii) excoriation and erosion, and (iv) scaling and dryness. The data analysis was carried out by blinded investigators.

### 4.5. Quantitative PCR

Total RNA samples were extracted from cells or mouse skin using Tri-RNA Reagent (Favorgen, Ping-Tung, Taiwan). First-strand cDNA synthesis from the total 500 ng of RNA was performed with PrimeScript RT master mix (RR036A, Takara, Shiga, Japan). The thermocycling condition was 15 min at 37 °C and 5 s at 85 °C. Synthesized cDNAs were subjected to real-time PCR with qPCR 2× Premix SYBR (Enzynomics, Korea) using a Stratagene Mx3000p qPCR machine (Agilent Technologies, Santa Clara, CA, USA). The PCR conditions used to amplify all genes were 10 min at 95 °C, 40 cycles of 95 °C for 15 s, and 64 °C for 40 se Expression data were calculated from the cycle threshold (Ct) values using the ΔCt method of quantification. *RPLP0* was used for normalization. Oligonucleotides are listed in Supplementary Table S1.

### 4.6. Immunocytochemistry and Histology

For immunocytochemistry, cells were fixed for 10 min with 4% paraformaldehyde in PBS and permeabilized with PBST solution (0.5% Triton X-100 in PBS) for 30 min. After blocking of cells with 5% BSA in PBST solution for 1 h, cells were incubated with anti-NF-κB p65 antibody (1:200, SC-372, Santa Cruz Biotechnology, Dallas, TX, USA) overnight at 4 °C. A secondary antibody conjugated to FITC (1:500, F0257, Sigma-Aldrich, St. Louis, MO, USA) was used. For histology, the harvested dorsal skin was immediately fixed with 4% paraformaldehyde in PBS and left overnight at 4 °C. The tissue was rinsed in PBS for 1 h and dehydrated through 25%, 50%, 70%, 90%, and 100% ethanol cycles for 1 h each, followed by 2 changes of 100% ethanol. The tissue was cleared through 2 changes of xylene for 1 h each and embedded in paraffin. The paraffin-embedded tissue block was sectioned at 5 μm and deparaffinized by 2 changes of xylene for 10 min each. Rehydration of sections was performed with 100%, 95%, 70%, and 50% ethanol in 5 min cycles. The sections were washed in tap-water for 5 min and stained with hematoxylin (DAKO, Glostrup, Denmark) for 10 min and dipped into acidic alcohol for 30 s. For counterstaining, the sections were stained with eosin (Millipore, Darmstadt, Germany) for 30 s and washed with tap water. Then, the sections were dehydrated in 70%, 95%, and 100% ethanol for 2 min and washed with xylene twice for 4 min. Lastly, the sections were mounted with mounting medium (Fluka, New Jersey, USA) and covered by coverslip. Mast cells were stained with toluidine blue (Sigma-Aldrich, St. Louis, MO, USA) without eosin staining.

### 4.7. Immunoprecipitation and Western Blot Analysis

For immunoprecipitation, cell extracts were prepared as described previously [83]. Briefly, HaCaT keratinocytes (1X 10^7^ cells) were lysed in 200 µL of IP150 buffer (25 mM Tris-HCl (pH 8.0), 150 mM NaCl, 0.1% NP-40, 0.5 mM EDTA, 10% glycerol) and sonicated using Bioruptor (KRB-01, CosmoBio, Tokyo, Japan) in 5 cycles, with 30 s sonication and 30 s rest for each cycle. After centrifugation, the supernatant was incubated overnight at 4 °C with anti-Fyn antibody (1 µg/mL, SC-365913, Santa Cruz Biotechnology, Dallas, TX, USA), followed by incubation with 20 µL of a 50% slurry of protein G agarose beads (Amicogen, Jinju, Gyeongsangnam-do, Korea) for an additional 6 h at 4 °C. After protein–antibody–protein G agarose beads were washed extensively with IP150 buffer, the protein–antibody complex was dissolved in an SDS sample buffer. Normal IgG (1 µg/mL, SC-2025, Santa Cruz Biotechnology, Dallas, TX, USA) was used as a control. Western blot analysis was carried out using anti-Fyn (1:1000, SC-365913, Santa Cruz Biotechnology, Dallas, TX, USA), anti-phospho-SRC family (Y416) (1:1000, AP0511, Abclonal, Woburn, MA, USA), anti-phospho SYK (Y525/526) (1:1000, 2710, Cell Signaling, Danvers, MA, USA), anti-SYK (1:1000, A2123, Abclonal, Woburn, MA, USA), anti-phospho IKK (S176/177) (1:5000, ab194528, Abcam, Cambridge, UK), anti-IKK (1:5000, ab178870, Abcam, Cambridge, UK), anti-phospho IκB (S32) (1:1000, 2859, Cell Signaling, Danvers, MA, USA), anti-REDD1 (1:5000, ab106356, Abcam, Cambridge, UK), anti-phospho mTOR (S2448) (1:1000, 2971S, Cell Signaling, Danvers, MA, USA), anti-phospho p70S6K (T389) (1:1000, 9205S, Cell Signaling, Danvers, MA, USA), anti-phospho 4EBP1 (S65) (1:1000, 9451S, Cell Signaling, Danvers, MA, USA), and anti-β actin (1:2500, 3C-47778, Santa Cruz Biotechnology, Dallas, TX, USA) antibodies. Mouse skin tissue was homogenized in 1 mL of Tri-RNA Reagent (Favorgen, Ping-Tung, Taiwan) using a tissue homogenizer. After addition of 200 μL of chloroform, the homogenates were centrifuged for 15 min at 12,000× *g* at 4 °C. The supernatant was removed and 500 μL of 100% ethanol was added to the interphase and lower organic phenol–chloroform phase, and these were centrifuged for 15 min at 12,000× *g* at 4 °C. The supernatant was transferred to a new microcentrifuge tube and the same volume of isopropanol was added and incubated for 10 min. After centrifugation at 12,000× *g* for 10 min, the resulting pellet was washed 3 times with 1 mL of 0.3 M guanidine hydrochloride in 95% ethanol. Each wash step included 20 min incubation and 5 min centrifugation at 7500× *g* at 4 °C. Then, the pellet was washed with 1 mL of 100% ethanol and centrifuged for 5 min at 7500× *g* at 4 °C. The washed pellet was sonicated in 1 M Tris-HCl (pH 8.0) buffer containing 100 μL of 8 M urea and 1% SDS (1:1 ratio), then centrifuged for 10 min at 3200× *g* at 4 °C. The supernatant was used for Western blot analysis. Data were quantitatively assessed and additionally depicted in graphs. All data represent means ± S.E.M.

### 4.8. Promoter Reporter Assay

HaCaT keratinocytes were transfected transiently using JetPRIME transfection reagent (114-07, Polyplus transfection, NY, USA) with NF-κB firefly luciferase reporter containing NF-κB binding sites (E8491, Promega, Madison, WI, USA) in conjugation with a control thymidine kinase-promoter-driven *Renilla* luciferase (E2241, Promega, Madison, WI, USA). Briefly, 1 µg of NF-κB luciferase reporter and 200 ng of *Renilla* luciferase reporter in 75 µL of DMEM were mixed with 2 µL of JetPRIME transfection reagent in 75 µL of DMEM. The transfection mixture was incubated for 15 min at room temperature and the entire volume was added to each well of a 12-well plate. After incubation for 48 h, cells were harvested and luciferase activity was measured using a dual luciferase reporter assay system (E1910, Promega, Madison, WI, USA). Briefly, cell culture media were removed and rinsed in 1X PBS. Cells were lysed in 200 µL of 1X PLB (Passive Lysis Buffer) for 15 min at room temperature. After centrifugation of cell lysate for 30 s, 20 µL of PLB lysate was mixed with 100 µL of luciferase activity reagent (LAR II) in a 96-well plate. Firefly luciferase activity was measured using a Lumat BL 9507 luminometer (Berthold Technologies, Bad Wildbad, Germany). Then, 100 µL of Stop and Glo assay reagent was added and the *Renilla* luciferase activity was measured. Th firefly luciferase activity of the gene promoter was normalized to *Renilla* luciferase activity.

### 4.9. Chromatin Immunoprecipitation (ChIP)

Chromatin immunoprecipitation (ChIP) was performed as described previously [83]. HaCaT keratinocytes (2 X 10^7^ cells) were cross-linked using 1% formaldehyde in PBS for 10 min at room temperature. To stop the cross-linking, cells were treated with 0.125 M glycine for 10 min. After washing with ice-cold PBS, cells were lysed with SDS lysis buffer (1% SDS, 10 mM EDTA, 50 mM Tris-HCl pH 8.0) and sonicated using a Bioruptor (KRB-01, CosmoBio, Tokyo, Japan) until DNA fragments were 500 bp in size. The extracts were centrifuged at 12,000 rpm for 10 min and the resulting soluble chromatin solutions were diluted 1:10 with ChIP dilution buffer (0.01% SDS, 1.1% Triton X-100, 1.2 mM EDTA, 16.7 mM Tris-HCl pH 8.0, 167 mM NaCl). Anti-NF-κB p65 (1 µg/mL, SC-372, Santa Cruz Biotechnology, Dallas, TX, USA) or normal IgG (1 µg/mL, SC-2027, Santa Cruz Biotechnology, Dallas, TX, USA) were added and incubated overnight at 4 °C, followed by 20 µL of a 50% slurry of protein G agarose beads for an additional 6 h at 4 °C. The beads were extensively washed with low salt wash buffer (0.1% SDS, 1% Triton X-100, 2 mM EDTA, 20 mM Tris-HCl (pH 8.0), 500 mM NaCl), high salt wash buffer (0.1% SDS, 1% Triton X-100, 2 mM EDTA, 20 mM Tris-HCl (pH 8.0), 150 mM NaCl), LiCl wash buffer (0.25 M LiCl, 1% NP-40, 1% deoxycholate, 1 mM EDTA, 10 mM Tris-HCl (pH 8.0)), and finally with TE buffer. After elution of the DNA–protein complex with 1% SDS, cross-linking was reversed with 200 mM NaCl for 4 h at 65 °C. The DNA was purified using a DNA extraction kit (Bionics, Seoul, Korea). Real-time PCR was performed with a Stratagene Mx3000P qPCR machine (Agilent Technologies, Santa Clara, CA, USA). Oligonucleotides are listed in Supplementary Table S2. The relative proportions of immunoprecipitated DNA fragments were determined using the Δ*C*t comparative method and normalized to input genomic DNA.

### 4.10. Preparation of CA-PH Beads

CA-PH beads were synthesized using amino-PEG1000-polystyrene resin (BeadTech, Ansan, Korea). The resin was swollen and incubated in *N*,*N*’-dimethylformamide (DMF) solution containing Fmoc-Photolabile linker (Creosalus), *N*,*N*,*N*’,*N*’-tetramethyl-*O*-(1H-benzotriazol-1-0yl)uranium hexafluoro-phosphate (HBTU), and *N*,*N*’-diisopropylethylamine (DIPEA) at room temperature for 2 h. To acetylate the remaining amine groups of the resin, the resin was treated with DMF containing acetic anhydride and DIPEA for 2 h. The loading level of the resulting resin (0.25 mmol/g) was confirmed by Fmoc titration after the linker coupling. The peptides were synthesized on the resin by repeating the coupling and deprotection steps: (a) in the coupling step, the resin was treated with DMF solution containing Fmoc amino acids (sequentially: 6-aminohexanoic acid, β-alanine, histidine, proline), HBTU, 1-hydroxybenzotriazole (HOBt), and DIPEA for 2 h; (b) in the deprotection step, the resin was treated twice with 20% piperidine–DMF (*v*/*v*) solution, first for 5 min and then for 10 min. The completion of each coupling reaction was confirmed by Kaiser’s ninhydrin test. For pre-activation, CA was dissolved in DMF, HOBt and DIC were added, and the mixture was stirred for 30 min at room temperature in the dark. After coupling of proline and removal of the Fmoc group, the resin was immersed in the HOBt-activated CA solution and DIPEA at room temperature for 2 h. The resin was washed with DMF, DCM, and MeOH three times after each coupling and deprotection step. The resin was immersed in methanol and the photolabile linker was cleaved from the resin by UV exposure at 365 nm for 15 min. The cleaved CA-PH was confirmed with HPLC (1260 Infinity, Agilent, Santa Clara, CA, USA) and a C18 reverse-phase column (AkzoNobel, Amsterdam, Netherlands).

### 4.11. Pull-Down Assay

HaCaT keratinocytes (1 × 10^7^ cells) were lysed in 200 µL of IP150 buffer and sonicated using a Bioruptor (KRB-01, CosmoBio, Tokyo, Japan) in 5 cycles, with 30 s sonication and 30 s rest for each cycle. After centrifugation, the supernatant was incubated with CA-PH coupled beads at 4 °C overnight in the absence or presence of 10 μl of 1 mM CA-PH. After beads were washed with 1 mL of IP150 buffer, CA-PH-bound protein was dissolved in an SDS sample buffer and Western blot analysis was performed using anti-Fyn antibody. The control was acetylated beads without CA-PH coupling.

### 4.12. Drug Affinity Responsive Target Stability (DARTS) Assay

Drug Affinity Responsive Target Stability (DARTS) assay was performed as previously described with slight modifications [84]. Briefly, HaCaT keratinocytes (1 × 10^7^ cells) were harvested and sonicated in 200 μL of IP150 buffer for 5 min. After centrifugation at 12,000 rpm for 10 min, the supernatants were added with 10× TNC buffer (50 mM Tris-HCl (pH 8.0), 50 mM NaCl, 10 mM CaCl_2_) and diluted to 1 mg/mL of protein. Different concentrations of CA-PH and 1 μg of Pronase (10-165-921-001, Roche, Basel, Switzerland) were added to the extract and incubated for 30 min at room temperature. The digestion reaction was stopped by adding SDS-PAGE sample buffer and Western blot analysis was carried out using anti-Fyn antibody. Then, β actin was used as a loading control.

### 4.13. Molecular Docking Prediction

Protein–ligand docking was performed using the Glide (Grid based LIgand Docking with Energetics) docking application in the Schrödinger suites program (Schrödinger LLC, NY, USA). The three-dimensional structures of Fyn protein (PDB ID: 2DQ7) and CA-PH (PubChem ID: 46911327) were prepared using Ligprep in Schrödinger suites. The receptor grid for Fyn was generated by specifying the binding (active) site residues, which were identified by SiteMap. SiteMap is a binding site prediction application in Schrödinger suites. After the receptor grid was generated, the ligands were docked to the Fyn using Glide docking protocol. The docked conformers were evaluated using the Glide (G) Score. The G Score is calculated as follows:

G Score = a*vdW + b*Coul + Lipo + Hbond + Metal + BuryP + RotB + Site

where vdW is the van der Waals energy, Coul is the Coulomb energy, Lipo is the lipophilic contact, HBond is hydrogen-bonding, Metal is metal-binding, BuryP is the penalty for buried polar groups, RotB is the penalty for freezing rotatable bonds, Site are polar interactions in the active site, and a = 0.065 and b = 0.130 are coefficients of vdW and Coul.

### 4.14. Kinase Assay and Enzyme Kinetic Study

Fyn, SYK, and JNK kinase activities were measured using the Kinase Enzyme System (V3571, V3801, V4070, Promega, Madison, WI, USA) and ADP-Glo Kinase assay (V6930, Promega, Madison, WI, USA) according to the manufacturers’ instructions. Briefly, 100 ng of Fyn, SYK, or JNK were incubated in reaction buffer A supplemented with 100 μM DTT, 0.2 μg of substrate peptide, 50 μM ATP, and the indicated concentration of CA-PH for 15 min at 37 °C. The reaction mixture was incubated with ADP-Glo Reagent for 40 min. Then, Kinase Detection Reagent was added and incubated for 30 min. Luminescence was detected using a Lumat BL 9507 luminometer (Berthold technologies, Bad Wildbad, Germany). For enzyme kinetic study, Fyn was incubated with various concentrations of ATP and CA-PH as indicated in Figure 3D. To determine of the type of inhibition, reciprocal plots (a plot of 1/enzyme velocity (1/V) versus 1/substrate concentration (1/[S])) were constructed using Lineweaver–Burk methods. Kinetic parameters were analyzed by Sigmaplot version 14.0 (Systat Software Inc., San Jose, CA, USA). The best kinetic model was judged by the highest R^2^ and Akaike information criterion correction (AICc) values [85] (Supplementary Table S3). The relationship of IC_50_ to *K_i_* is given by IC_50_ = *K_i_*(1 + [S]/*Km*) [86].

### 4.15. Statistical Analyses

All quantitative data are presented as means ± S.E.M. for three independent experiments. The differences between two groups were evaluated by a paired t-test. Analysis of variance (ANOVA) was used for multiple comparisons. Significance values were * *p* ≤ 0.05, ** *p* ≤ 0.01, and *** *p* ≤ 0.005.

## Supplementary Materials

Supplementary materials can be found at https://www.mdpi.com/1422-0067/21/19/7160/s1.

## Abbreviations

CA-PH	caffeoyl-prolyl-histidine amide
AD	atopic dermatitis
DNFB	1-fluoro-2,4-dinitrobenzene
NF-κB	nuclear factor kappa-light-chain enhancer of activated B cells
SYK	spleen-associated tyrosine kinase
JNK	c-JUN N-terminal kinase
REDD1	regulated in development and DNA damage response 1
DARTS	drug affinity responsive target stability
ChIP	chromatin immunoprecipitation
