# Supplementary material for: Caffeoyl-Prolyl-Histidine Amide Inhibits Fyn and Alleviates Atopic Dermatitis-Like Phenotypes via Suppression of NF-κB Activation
Source: Int J Mol Sci. 2020 Sep 28;21(19):7160. doi: 10.3390/ijms21197160 (PMC7582254; doi:10.3390/ijms21197160)
Supplement: Supplementary file 1 [file ijms-21-07160-s001.pdf]

Supplementary Materials.

Supplementary Figures

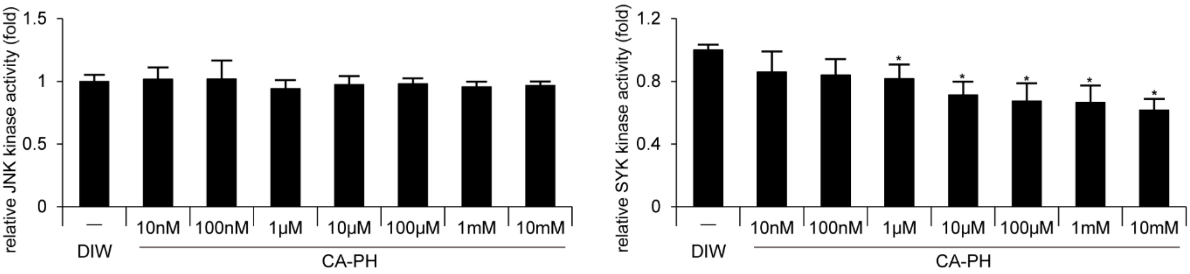

Figure S1. CA-PH does not inhibit JNK or weakly inhibits SYK. JNK or SYK activity was measured at the indicated concentrations of CA-PH (n=3). Kinase assay was performed using Kinase Enzyme System. All data represent mean ± S.E.M. Significance value was * $P \leq 0.05$.

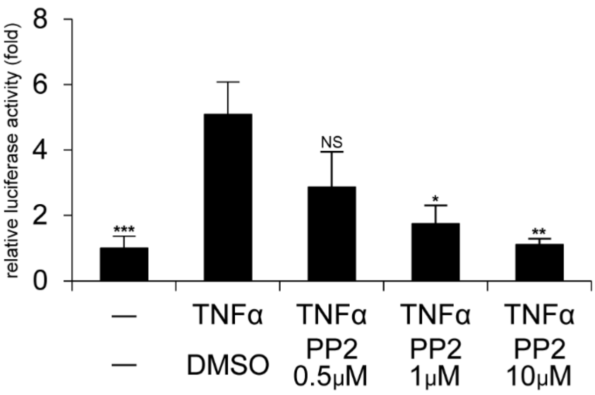

Figure S2. PP2, an inhibitor for Src-family kinases including Fyn, suppressed activity of promoter reporter containing NF-κB binding sites in TNFα-treated HaCaT keratinocytes. DMSO was used as solvent for PP2. Following transfection of luciferase reporter vector containing the NF-κB binding site and control *Renilla* luciferase expression vector into HaCaT keratinocytes, luciferase activity was measured in cell extracts (n=5). Reporter activity is represented as fold activation relative to *Renilla* luciferase activity. All data represent mean ± S.E.M. Significance values were * $P \leq 0.05$, ** $P \leq 0.01$, and *** $P \leq 0.005$.

Supplemental Tables

Table S1. Oligonucleotide primers used for quantitative PCR in this study.

Gene	Forward	Reverse
**hRPLP0**	AGCCCAGAACACTGGTCTC	ACTCAGGATTTCAATGGTGCC
**hTSLP**	TATGAGTGGGACCAAAAGTACCG	GGGATTGAAGGTTAGGCTCTGG
**hIL-10**	TCAAGGCGCATGTGAACTCC	GATGTCAAACTCACTCATGGCT
**hIL-13**	GAGGATGCTGAGCGGATTCTG	CACCTCGATTTTGGTGTCTCG
**hIL-25**	CAGGTGGTTGCATTCTTGGC	GAGCCGGTTCAAGTCTCTGT
**hREDD1**	TGGGCAAAGAACTACTGCG	AGAGTTGGCGGAGCTAAACAG
**mRPLP**	AGATTCGGGATATGCTGTTGGC	TCGGGTCCTAGACCAGTGTTC
**mTSLP**	ACTGCAACTTCACGTCAATTACG	TTGCTCGAACTTAGCCCCTTT
**mIL-10**	GCTCTTACTGACTGGCATGAG	CGCAGCTCTAGGAGCATGTG
**mIL-13**	CCTGGCTCTTGCTTGCCTT	GGTCTTGTGTGATGTTGCTCA
**mIL-25**	ACAGGGACTTGAATCGGGTC	TGGTAAAGTGGGACGGAGTTG
**mREDD1**	TGGTGCCCACCTTTCAGTTG	GTCAGGGACTGGCTGTAACC

Table S2. Oligonucleotide primers used for ChIP in this study.

Binding Sites	Forward	Reverse
**NF-κB #1 (hTSLP)**	TGAGCATATGAAAACCAAGAAG	TTGAAAAATAGTTGCCAAAAGGA
**NF-κB #2 (hTSLP)**	GGGCAAAGCAAAAAGGAGGAAG	TAAACGCCTACGGGCTCTTT
**NF-κB (hIL-10)**	GGGAAGGTGAAGGCTCAATCA	GTGTTGCTCTACCTGGGGAA
**NF-κB (hIL-13)**	TGATCCTGCAGAGACTGGTGA	GTATGGGCCATCCTTCTGGGA
**NF-κB (hIL-25)**	TGGACCTGCTGAGGCTGAGT	GTTGGCTGGATGGGCAAGGT

Table S3. R^2 and AICc values analyzed by SigmaPlot.

Rank by AICc	Equation R^2	AICc
**Competitive (Full)**	0.99418	-134.083
**Mixed (Full)**	0.99418	-129.719
**Noncompetitive (Full)**	0.9902	-125.75
**Uncompetitive (Full)**	0.98316	-117.096
